# CO-PRODUCTION TO TAILOR A DIGITAL TOOL FOR MONITORING SYMPTOMS OF DEMENTIA IN NURSING HOME CARE IN SWEDEN

**DOI:** 10.1093/geroni/igad104.2404

**Published:** 2023-12-21

**Authors:** Johannes Malm, Therése Bielsten, Elzana Odzakovic, Deborah Finkel, Charlotta Nilsen, Ingemar Kåreholt

**Affiliations:** Institute for Gerontology, Jönköping, Jonkopings Lan, Sweden; Institute of Gerontology, Jönköping, Jonkopings Lan, Sweden; Institute of Gerontology, Jönköping, Jonkopings Lan, Sweden; Jönköping University, Jönköping, Jonkopings Lan, Sweden; Institute of Gerontology, Jönköping, Jonkopings Lan, Sweden; Jönköping University, School of Health and Welfare, Jönköping, Jonkopings Lan, Sweden

## Abstract

Symptoms of dementia change over time, resulting in complex situations that can negatively impact the person with dementia, as well as their relatives, and create challenges for staff members. Behavioral and Psychological Symptoms of Dementia (BPSD), such as delusions, hallucinations, agitation, depression, anxiety, apathy, irritability, aberrant motor behavior, and sleep disturbances, occur in approximately 90% of older people with dementia. The purpose of the study was to identify potential barriers and facilitators prior to introducing a web-based digital tool, the Daily-BPSD. Daily-BPSD is going to be used by staff members for daily registrations of severity levels of BPSD for persons with dementia in nursing home care in Sweden. Qualitative data collection was conducted in co-production with previous users of a similar digital tool in disability care (n = 11) and future users in dementia care (n = 32). The participants were assistant nurses, care managers, nurses, occupational therapists, and relatives. The responses highlight the importance of an accessible and time-effective registration procedure, a manageable number of variables and registrations occasions per day, and ensuring that the same information does not need to be documented in different systems. The findings will be used to tailor Daily-BPSD and adequately prepare staff members for large-scale data collection in the next step of the research project. Daily-BPSD could provide an extended foundation of knowledge of the person with BPSD, which could be used to provide more person-centered and appropriate care.

